# Palette of Luciferases: Natural Biotools for New Applications in Biomedicine

**DOI:** 10.32607/actanaturae.10967

**Published:** 2020

**Authors:** A. A. Kotlobay, Z. M. Kaskova, I. V. Yampolsky

**Affiliations:** Shemyakin-Ovchinnikov Institute of Bioorganic Chemistry of the Russian Academy of Sciences, Moscow, 117997 Russia; Pirogov Russian National Research Medical University, Moscow, 117997 Russia

**Keywords:** bioluminescence, luciferase, bioluminescent systems

## Abstract

Optoanalytical methods based on using genetically encoded bioluminescent enzymes, luciferases, allow one to obtain highly sensitive signals, are non-invasive, and require no external irradiation. Bioluminescence is based on the chemical reaction of oxidation of a low-molecular-weight substrate (luciferin) by atmospheric oxygen, which is catalyzed by an enzyme (luciferase). Relaxation of the luciferin oxidation product from its excited state is accompanied by a release of a quantum of light, which can be detected as an analytical signal. The ability to express luciferase genes in various heterological systems and high quantum yields of luminescence reactions have made these tools rather popular in biology and medicine. Among several naturally available luciferases, a few have been found to be useful for practical application. Luciferase size, the wavelength of its luminescence maximum, enzyme thermostability, optimal pH of the reaction, and the need for cofactors are parameters that may differ for luciferases from different groups of organisms, and this fact directly affects the choice of the application area for each enzyme. It is quite important to overview the whole range of currently available luciferases based on their biochemical properties before choosing one bioluminescent probe suitable for a specific application.

## INTRODUCTION

Modern biomedical research, which includes high-throughput drug screening, detailed studies of the mechanisms of disease development, and design of new instruments for personalized medicine, relies on various analytical methods including bioimaging.

**Fig. 1:**
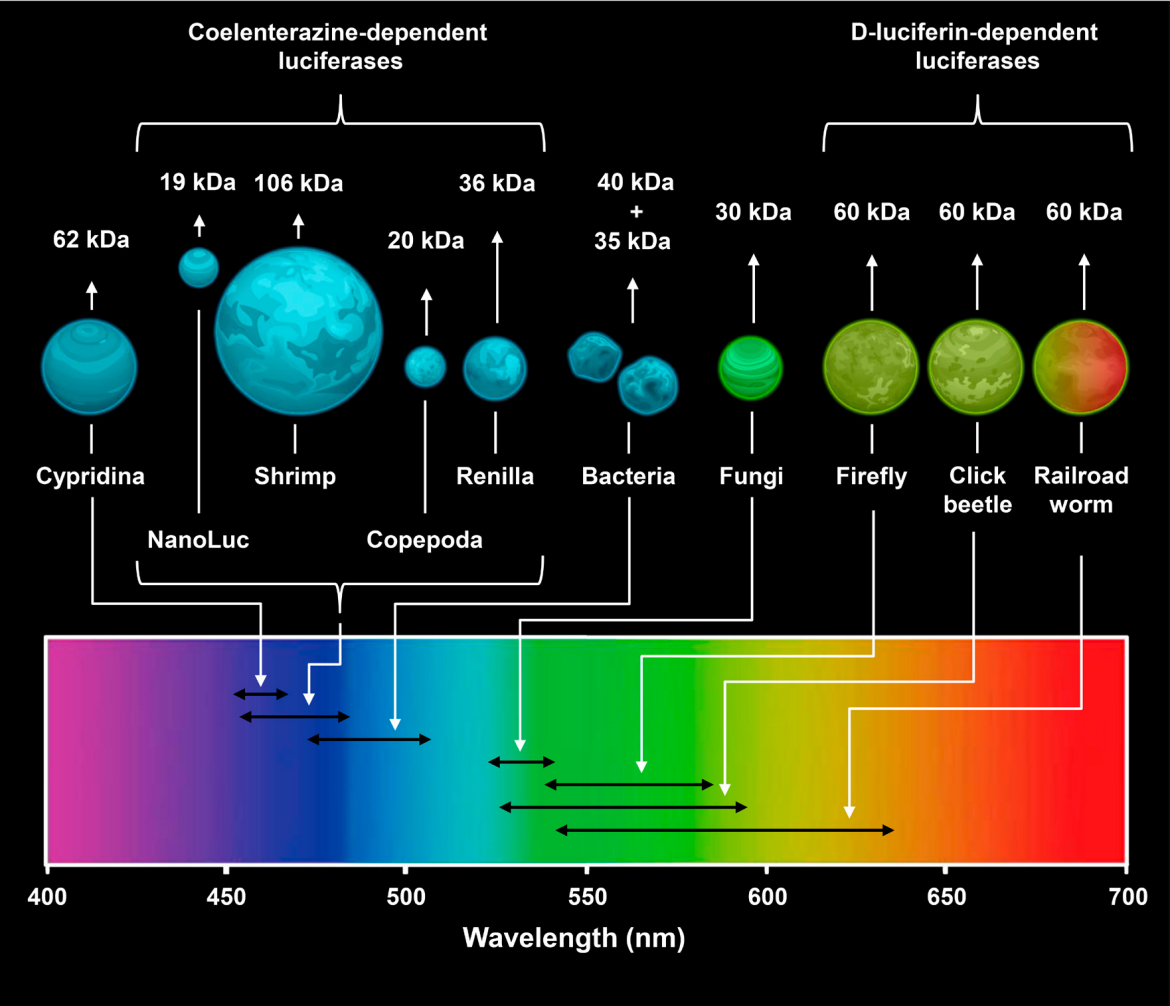
Key properties of natural luciferases. Average size and spectral characteristics of natural luciferases from different organisms and NanoLuc

A wide range of physicochemical methods are used in modern science and medicine for bioimaging, realtime non-invasive visualization of biological processes [1]. The optical bioimaging methods based on genetically encoded instruments, such as fluorescent proteins and bioluminescent luciferases (*Fig. 1*), allow one to obtain highly sensitive (down to the level of a single cell) and precise analytical signals from living tissues and organisms [2]. Bioluminescent methods are superior to the fluorescent ones as they require no excitation, which is often toxic to living cells, and there is no interference from light scattering or autofluorescence. All these factors ensure higher sensitivity. In addition, luciferases do not exhibit photobleaching, which is characteristic of fluorescent probes. Bioluminescence provides good spatial resolution and simple signal quantification.

**Fig. 2:**
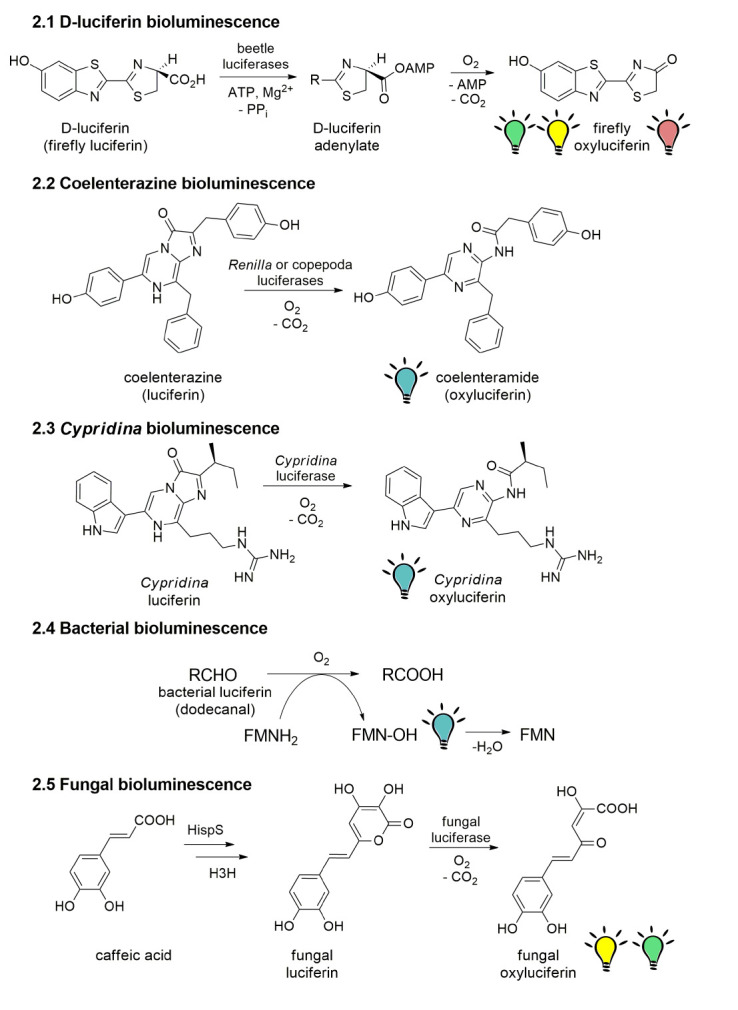
Mechanisms of bioluminescence.** 2.1 **Structure of *D*-luciferin and mechanism of its bioluminescence.** 2.2 **Reaction of coelenterazine bioluminescence.** 2.3 **Structure of cypridinid luciferin and its bioluminescence reaction.** 2.4 **Scheme of bacterial bioluminescence. RCHO – bacterial luciferin (dodecanal); RCOOH – bacterial luciferin oxidation product; FMNH_2_ – flavin mononucleotide (riboflavin-5′-phosphate) reduced form (cofactor); FMN-OH – FMN-4a-hydroxide, light-emitting substance; FMN - flavin mononucleotide oxidized form.** 2.5 **Scheme of fungal bioluminescence starting from caffeic (3,4-dihydroxycinnamic) acid

Bioluminescence (BL), or glowing of living organisms, is based on oxidation of a low-molecular-weight substrate, luciferin, by oxygen, with the reaction being catalyzed by an enzyme called luciferase. From approximately 40 different mechanisms of BL that currently exist, only 10 are studied in different degrees of depth. Five of them (*Fig. 2*) have already found applications in numerous analytical methods. The main purpose of this review is to describe the diversity and features of natural luciferases, which could be used for the development of novel bioimaging and other analytical methods in the field of biomedicine.

Format of the current review does not allow to consider the whole palette of natural luciferases that are theoretically available for practical use. There are several fairly well-studied mechanisms of BL, for which applications are still rather limited. For example, photoproteins, which utilize substrate in the activated form (2-hydroperoxycoelenterazine) non-covalently bound to the protein hydrophobic cavity [3], emit a characteristic brief flash of blue light; however, the regeneration of the enzyme-substrate complex can take several hours [4]. This is a major disadvantage for application of photoproteins along with the dependence on calcium ion concentration. Prospects for use of marine polychaete *Odontosyllis *and dinoflagellate bioluminescent systems remain uncertain as their luciferins are rather unstable and still not synthetically available [5, 6].

The biomedical research methods based on the luciferin–luciferase reactions play a significant role in modern science. The range of their applications is enormous: from analytical *in vitro *and *in vivo *methods to real-time bioimaging of living systems [2]. However, several drawbacks limit the use of luciferases and encourage further studies focused on natural bioluminescent systems in order to search for new luciferins and luciferases to broaden the range of methods and improve the existing analysis tools.

## 1. D-LUCIFERIN-DEPENDENT LUCIFERASES

Among insects, bioluminescent species are present in four orders: Hemiptera, Coleoptera, Diptera, and Collembola. However, current biochemical and molecular studies are generally focused on representatives of Coleoptera and Diptera. Dipteran BL, in contrast to coleopteran, has been scarcely studied. The order of Coleoptera comprises three families with bioluminescent properties, including fireflies (Lampyridae), click beetles (Elateridae), and railroad worms (Phengodidae). Bioluminescent systems of all the investigated coleopteran species depend on the common substrate first discovered in fireflies – *D*-luciferin. The BL reaction catalyzed by the firefly luciferase occurs in two steps: adenylation of *D*-luciferin and oxygenation of adenyl-luciferin (*Fig. 2.1*). For adenylation, presence of ATP and Mg^2+^ cofactors is necessary.

**1.1 Firefly luciferases**

At present, luciferase-encoding genes from a range of firefly species are known. In general, firefly luciferases are monomeric euglobulins of 60 kDa that are prone to dimerization in concentrated solutions [7]. Amino acid sequences of luciferases from different firefly species demonstrate 60–80% identity [8]. Additionally, the firefly luciferases have two independent binding sites for ATP and *D*-luciferin on their surface, as well as a binding site for D-luciferyl adenylate [9].

**Fig. 3:**
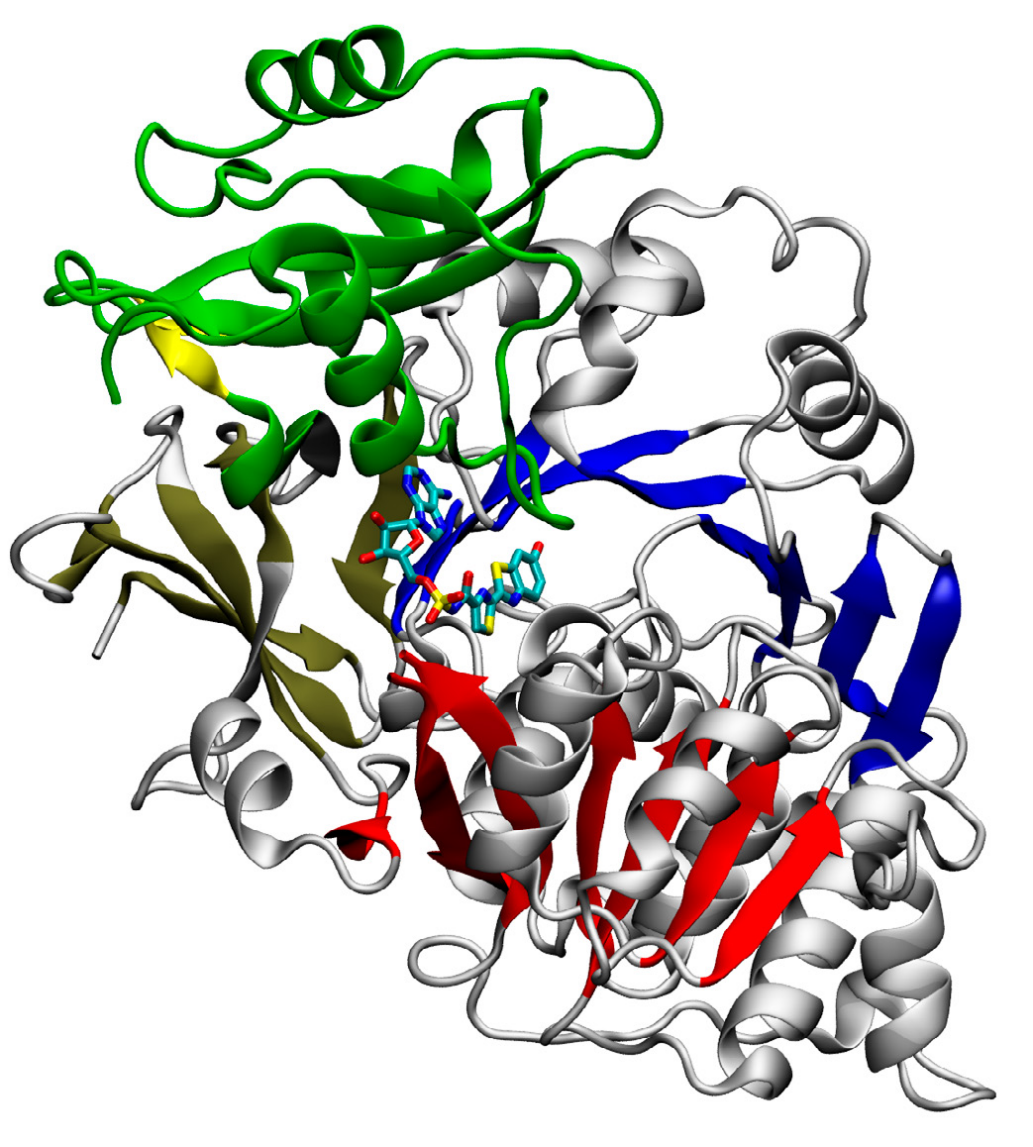
Crystal structure of the wild-type *P. pyralis *luciferase in the adenylate-forming conformation bound to DLSA

The first crystal structure of firefly luciferase (free enzyme) was reported in 1996 [10]. Over the past decades, crystal structures of luciferase at various catalytic stages, including in adenylated form (with DLSA) and in oxidative (with luciferyl-adenylate) and post-reaction (with AMP/oxyluciferin complex) conformations, have been reported [11,12]. The structures obtained supported the role of luciferase in BL color modulation and provided insights into the biochemical mechanism of the oxidative step of luciferase reaction. This data stimulated further detailed studies of the enzyme resulting in a great influx of new structural information, surpassing all the data obtained for any other luciferase. As can be seen in *Fig. 3*, the protein is composed of two globular domains: larger N-terminal domain and smaller C-terminal domain with a peroxisomal targeting signal. The tertiary structure of firefly luciferase consists of two β-strands flanked by α-helices, together forming an αβαβα motif and a β-barrel. The active site of the enzyme is formed with the surfaces of N- and C-terminal domains facing each other. During BL reaction, firefly luciferase undergoes considerable conformational change and the N- and C-terminal domains come close enough to sandwich the substrates [10]. The C-terminal domain has been shown to determine the firefly luciferase activity (deletion of its last 12 amino acids leads to complete loss of BL) [13].* D*-luciferin binding sites have also been identified [14]. The information obtained has led to the development and successful use of genetically modified luciferases with improved properties.

The quantum yield of firefly BL was first estimated to be 88 ± 25% by Seliger H.H. and McElroy W.D. in 1960 [15]. Later, the maximum quantum yield of *Photinus pyralis *BL was recalculated by Ando Y. et al. and was found to be 41.0 ± 7.4% at pH 8.5, with the yield decreasing with decrease in pH [16].

The effect of bivalent ions on firefly BL has also been studied [17]. Studies have shown that increase in Mn^2+^, Ca^2+^, or Mg^2+^ concentrations does not change the quantum yield or emission color, while the presence of Zn^2+^, Cd2+ Fe^2+^, Ni^2+^, and Co^2+^ ions induces a bathochromic shift [17,18]. The quantum yield of the BL reaction shows highest sensitivity to Hg^2+^ ions. Increase in Hg^2+^ concentration induces a sharp decrease in the quantum yield of the reaction.

One of the most important parameters for practical application of luciferases is the wavelength of maximum of BL emission. For different species of fireflies and other *D*-luciferin utilizing organisms, natural emission maxima range from green (534 nm) to red (638 nm) [8]. It was shown that the color of firefly BL undergoes a bathochromic shift with decrease in pH [15, 16], increase in temperature, or in the presence of bivalent metal ions [17, 18]. At the same time, it has been observed in various *in vitro *experiments that the color of BL for luciferases of other Coleoptera does not depend on the abovementioned reaction conditions [17, 19].

Despite the intensive studies of the mechanism of BL color modulation, the chemical basis of the process and specific active site interactions remain unresolved. Evidently, the color of BL depends on two main factors, including the structure of the light emitter and amino acid residues at the active site of luciferase, which form the micro-environment for the emitter. According to different studies, one of the hypotheses states that luminescence color is determined by active site conformation, which indirectly affects polarity and specific interactions around oxyluciferin [20]. A closed nonpolar conformation would correspond to green light emission and an open and/or more polar conformation would result in red luminescence [21, 22].

A variety of stable mutant forms of firefly and other coleopteran luciferases with BL colors such as yellow-green, red, and even near infrared is currently available. A modification in *D*-luciferin is an alternative approach for changing the wavelength of BL maximum. To date, a wide range of *D*-luciferin analogs that are able to induce a spectral shift in beetle luciferase luminescence, including NIR wavelength range, has been developed [23-25].

Another factor to be considered while developing new applications for natural luciferases is their limited thermostability range. The majority of these enzymes are inactivated at even moderate temperatures (30°C), which plays a crucial role in their *in vivo* applications. The brightness of BL reaction, which is a function of quantum yield, K_m_, V_max_, turnover rate, protein stability, and sensitivity to product inhibition [26], is another important parameter that has to be considered, for example, in microscopy. In order to overcome the drawbacks of natural enzymes, several brighter analogs of firefly luciferase and luciferases with increased thermostability have been generated with the help of site-directed mutagenesis methods [27].

Thus, some properties of firefly luciferases, such as high quantum yield, diverse color palette of BL, and unique mechanism of its color modulation, make these enzymes a very effective tool for biotechnology. Conversely, the requirement of cofactors (ATP, Mg^2+^) and sensitivity of the BL spectra to pH value, bivalent metal ions and temperature, may, in some cases, become a disadvantage. Nevertheless, despite all the limitations, firefly bioluminescence system is now widely used in numerous branches of science, and its practical potential has not exhausted yet. Various chimeric constructs and thermostable, chemoresistant luciferases, as well as luciferases with shortened intracellular half-life have been developed based on firefly luciferase and they have been described in corresponding reviews [28, 29].

**1.2 Click beetle luciferases**

The bioluminescent system of click beetles, which also utilizes *D*-luciferin as a substrate, is fairly well-studied. Several luciferases from different species of Elateridae family have been identified, cloned, and characterized. These proteins have a molecular weight of approximately 60 kDa. Click beetle BL peaks in the range from 532 to 593 nm [30]. However, the value of this parameter can differ even for insects of the same species living in different populations [31].

The first click beetle bioluminescent system studied was the system of Jamaican *Pyrophorus plagiophthalamus*. Four types of luciferases possessing different colors of BL were cloned from one organism (from head spots and abdominal light organs): green (546 nm), yellow- green (560 nm), yellow (578 nm), and orange (593 nm) [32]. cDNAs encoding these four luciferases have shown high degree of homology between the proteins (from 95 to 99%), while the homology with firefly luciferase was much lower (about 47%) [32, 33]. Like firefly luciferase, these enzymes have a peroxisomal targeting signal at the C-terminus.

Color variability and pH-insensitivity of click beetle luciferases within the physiological range of pH (from 6 to 8) make them a rather attractive choice for *in vivo *analytical methods. Green and red forms of *P. plagiophthalamus *luciferase and their genes are commercially available (CBG – green form and CBR – red form). In addition, these luciferases are the smallest among insect luciferases (about 543 amino acids). However, they are prone to aggregation and form active dimers in concentrated solutions [9], which should be taken into account before planning *in vivo *experiments.

Increase of signal intensity of click beetle BL is the focus of research for several scientific groups. For example, a mutant of click beetle luciferase, which is ten times brighter than the natural firefly luciferase, was developed for use in bioimaging [34]. Influence of amino acid composition on the color of BL for clickbeetle luciferases has been studied by Viviani V.R. and colleagues [35].

**1.3 Railroad worm luciferases**

Currently, luciferases from only four Phengodidae species have been cloned and studied. Among them, the BL of *Phrixothrix vivianii *is probably the most studied. There are two different luciferases found within one organism of this species with considerably different BL spectra with λ_max_ = 542 nm (yellow- green) and λ_max_ = 620 nm (red) [36, 37]. Meanwhile, the bioluminescent system of *Phrixothrix hirtus *has the most “red” emission (λ_max_ = 636 nm) among all Coleoptera [37].

Biochemical properties of railroad worm luciferases have been studied poorly. Similarly to click beetle luciferases, the maximum of their BL is pH-insensitive [19, 38]. In a study, two luciferases from *Phrixothrix vivianii *were cloned – Pv_GR_ (λmax = 542 nm) and Ph_RE_ (λmax = 622 nm) [37]. Both have molecular weight of about 60 kDa. Their amino acid sequences have quite a high degree of homology with each other (71%) and with corresponding luciferases from the Japanese railroad worm *Rhagophthalmus ohbai *(66.6% for Pv_GR_ and 56% for Ph_RE_), which is quite common for related species [39]. However, the homology of Pv_GR_ and Ph_RE_ with Lampyridae (50–55% and 46–49%, respectively) and Elateridae luciferases (47–49%) is comparatively lower, which signifies that these enzymes evolved independently [37]. The click beetle luciferases also contain a tripeptide at their C-terminus, which is responsible for their localization in peroxisomes [7].

## 2. COELENTERAZINE-DEPENDENT BIOLUMINESCENT SYSTEMS

Marine organisms comprise a significant number of all known bioluminescent species. For most of them, the bioluminescent substrate is celenterazine (*Fig. 2.2*) [40], including soft corals (*Renilla*), copepods, ostracods (*Conchoecia*), cephalopods (*Vampyroteuthis*), scyphozoan jellyfish (*Periphylla*), and decapods (*Oplophorus*).

All coelenterazine-dependent luciferases can be divided into two groups. One of the two groups consists of “true” luciferases, which catalyze a typical luciferin-luciferase reaction resulting in formation of oxyluciferin, which emits a quantum of light (*Fig. 2.2*). Another group contains photoproteins - bioluminescent proteins, which have not been considered in this review.

**2.1 Soft coral** *Renilla* **luciferases**

At present, the sequences of *Renilla reniformis *and* Renilla muelleri *luciferases (RLuc) are known [41].* Renilla reniformis *luciferase has molecular weight of 36 kDa. RLuc is the only intracellular luciferase among all the coelenterazine-dependent luciferases. Besides luciferase, coelenterazine, and oxygen, *Renilla *BL system requires two supplementary proteins: coelenterazine- binding protein (CBP) and green fluorescent protein (GFP) [42, 43]. RLuc is able to catalyze *in vitro* chemiluminescence of coelenterazine without the need for additional proteins; however, in the presence of GFP, this reaction proceeds with a much higher quantum yield. The formation of RLuc-GFP complex has been proven experimentally [43]. Maximum of luminescence in this case is red-shifted (from 480 to 509 nm) due to bioluminescence resonance energy transfer (BRET).

**Fig. 4:**
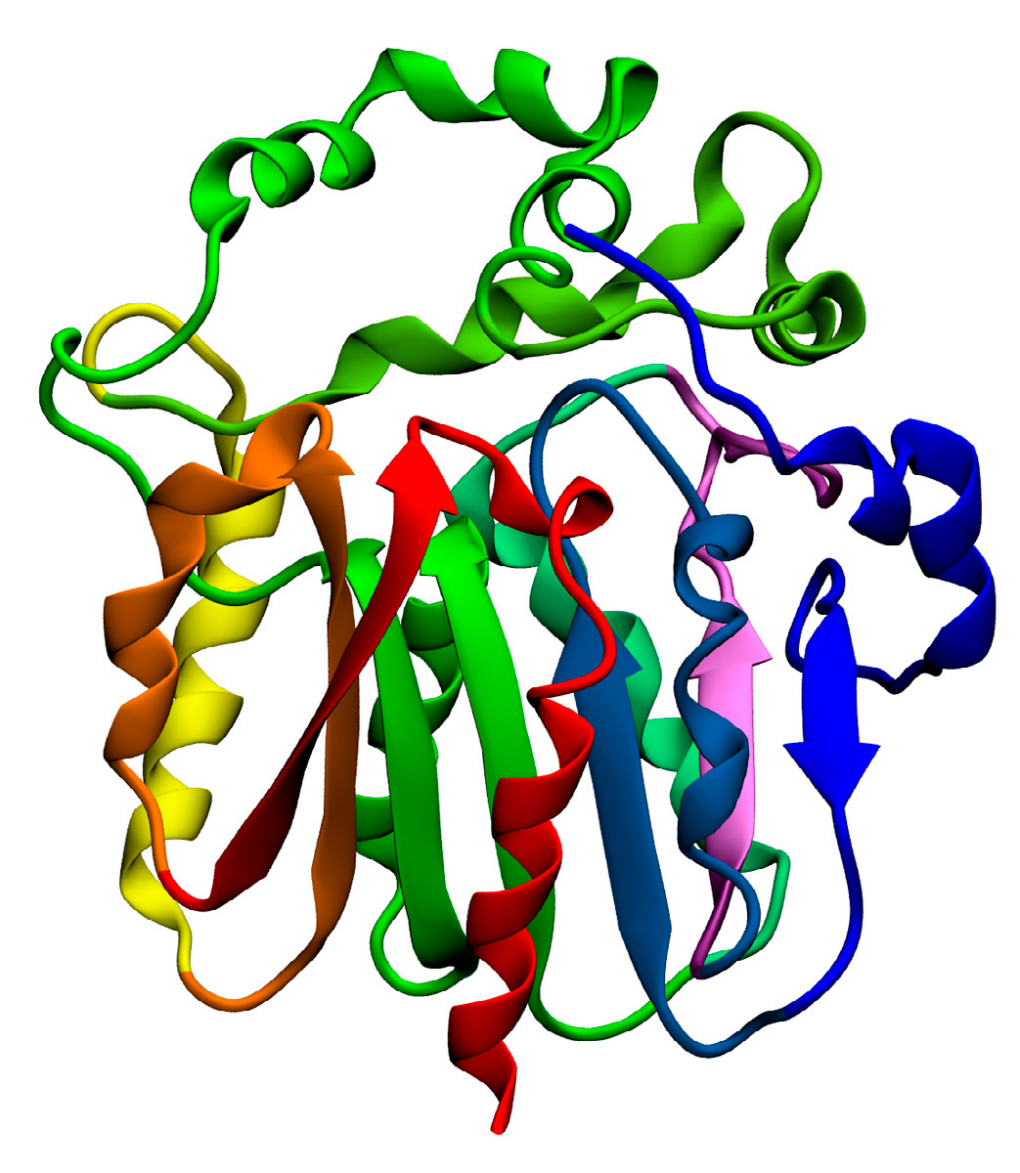
Structure of *R. reniformis *luciferase

The amino acid sequence of RLuc shows no significant relationship with other coelenterazine-dependent luciferases, but reveals similarities with α/β hydrolase family proteins [44]. This data was obtained from the crystal structure of *Renilla* luciferase [45] (*Fig. 4*).

Natural RLuc possesses biochemical properties that make it suitable for different analytical applications. The temperature optimum for enzyme activity is 18–37°C, the pH optimum is between 6.0 and 7.0, but the quantum yield of BL reaction is rather low (5.3%) [46]. These properties make RLuc one of the most favorable reporters in cellular research and *in vitro* analysis. Blue emission and low quantum yield of BL limit the use of RLuc in *in vivo *assays [44], as animal tissues significantly absorb visible light outside the “transparency window” (600–00 nm). To overcome these limitations, a number of RLuc-based reporters with improved properties have been obtained using random or site-directed mutagenesis, such as mutants with increased resistance to inactivation by blood serum, enhanced brightness, or proteins with red-shifted spectra [44, 47, 49, 49]. To expand the scope of applications of RLuc, several coelenterazine analogs with increased brightness of luminescence and red-shifted emission have also been generated [49, 50].

**2.2 Luciferases of Copepoda**

Twenty eight sequences of luciferases from representatives of 12 different species of copepods (subclass Crustacea) are currently known. Some of them have several genes encoding up to three luciferase isoforms. Interestingly, the homology between luciferase isoforms from one copepod species is comparable to that between luciferases from different, often taxonomically distant, species [51]. Despite the fact that copepod luciferases are widely used in various studies both* in vitro *[52, 53] and *in vivo *[54, 55], their native structure is still unknown.

The first luciferases to be cloned were those of* Gaussia princeps *(GLuc) [56] and *Metridia longa* (MLuc) [55]. These are small, about 20 kDa, secreted proteins. The copepod luciferase consists of a signal peptide essential for secretion, a variable N-terminal domain, and a conservative C-terminal domain. Apparently, the variable domain is not directly related to the BL function of the enzyme; moreover, its absence in creases the rate of BL reaction. Mutant forms of MLuc have approximately 1.5–3 times higher luminescent activity than full length luciferases [57]. The conservative domain of copepod luciferase consists of two non-identical tandem repeats of 70 amino acids, each containing a highly conservative fragment of 32 amino acids [55, 58]. Data on the effect of these repeats on bioluminescent activity is very contradictory. According to some reports, expression of one of these tandem repeats in *E. coli *induced BL [58], although this was not confirmed by similar experiments in eukaryotic expression systems [59].

In general, expression of recombinant copepod luciferases in *E. coli *is quite problematic because of considerable aggregation of recombinant proteins, resulting in heterogeneity of the final sample [52, 60]. In addition, copepod luciferases contain up to five disulfide bonds; however, the redox potential in the cytoplasm of bacterial cells does not facilitate their formation. Therefore, the bioluminescent activity of recombinant luciferases obtained by their bacterial expression is several times lower than those expressed in insect cells [60, 61, 62]. The first highly-active, monomeric MLuc protein (MLuc7 isoform) refolded from *E. coli *inclusion bodies was obtained recently [63]. However, using the secreted form of luciferase with KDEL sequence (this signal retains the protein in the endoplasmic reticulum) at the C-terminus can significantly increase its bioluminescent intensity within cells [54].

Natural luciferases from copepods possess extreme thermostability [64]. Even after being subjected to boiling for one hour, the isoform MLuc7 loses only 50% of its activity [61]. The luciferin-luciferase reaction of copepods is relatively faster than that of other coelenterazine- dependent luciferases [52, 54, 57], which may not be suitable for some applications. However, since copepod BL is highly dependent on buffer composition, the rate of reaction may be decreased by addition of detergents to the reaction mixture [65], though it is not possible for *in vivo *experiments.

As secreted proteins, natural copepod luciferases are most effective in studies of extracellular processes, intercellular interactions, and in bioimaging of intact tissues or small laboratory animals. A linear correlation between intensity of BL signal in culture medium and number of cells secreting GLuc [54, 66] and MLuc [67] reporter proteins has been proved. Therefore, the use of these luciferases is popular in methods concerning the functional state of malignant tumors, including the rate of their growth and metastasis, as well as their response to therapy, which could be assessed by the level of bioluminescent activity in blood samples [66, 67]. Other traditional applications of copepod luciferases could be found elsewhere [68].

Small size, stability, and high BL intensity of copepod luciferases inspire the design of novel applications [69]. Secretion signal makes them suitable for real time *ex vivo *monitoring of biological processes in medium of cultured cells and blood or urine in animals. New GLuc mutants displaying a 10-fold greater intensity relative to the parent luciferase [70] and glow-type light emission kinetics [65], and miniature 16.5 kDa [61], psychrophilic, and thermostable isoforms [71] of MLuc have been developed. These proteins open up new possibilities for implementation of copepod luciferases in research. Meanwhile, signal quenching, absorption of blue light *in vivo*, and rapid light decay of natural luciferases might complicate their use.

**2.3** *Oplophorus gracilirostris* **luciferase**

First samples of coelenterazine-dependent *O. gracilirostris* luciferase (OLuc) were characterized in 1976 [72]. The molecular weight of OLuc is about 106 kDa [73]. *Oplophorus *BL has an emission maximum at 454 nm and its brightness is strongly influenced by temperature, pH, and salt concentration. The temperature optimum of *Oplophorus *BL reaction is approximately 40°C and pH optimum is at pH 9 (the luciferase loses its bioluminescent activity at acidic pH). OLuc molecule is composed of four subunits: two with molecular weight of 19 kDa and the other two with molecular weight of 35 kDa. Only the 19 kDa protein subunits demonstrate BL activity, which is significantly lower than that of the natural luciferase [74]. This fact indirectly shows that the role of larger subunit is stabilization of the catalytic fragment in natural enzyme.

Computer modeling of secondary and tertiary structure of proteins and protein domains showed that* Oplophorus *luciferase was closely related to a group of membrane lipid-binding proteins. This allowed the researchers to obtain a mutant form of the 19 kDa protein subunit with 3 times higher BL activity and 1.5 times higher stability compared to the original enzyme, by a single amino acid substitution at position 166 [75]. This mutant was further transformed into a form called NanoLucR (NLuc) using three rounds of random mutagenesis. Thermostable NLuc has 16 amino acid substitutions and demonstrates much better characteristics compared to the wild type protein. The brightness of the BL reaction of NLuc with furimazine (coelenterazine analog) in lysates of HEK293 cells was 2.5 million times higher than that of the reaction of 19 kDa wild type luciferase and coelenterazine in the same conditions, and 150 times higher than that of the BL reaction of firefly luciferase or *Renilla *luciferase in similar conditions [75]. However, it should be mentioned that the increase in NLuc BL activity was significantly lower in similar experiments performed using lysates of *E.coli* and CHO cells [76].

## 3. LUCIFERASES OF Cypridina CRUSTACEANS

A unique bioluminescent system based not on coelenterazine, but on a luciferin having a different structure, was found in a crustacean belonging to the genus* Cypridina*. The structure of the luciferin from *Cypridina (Vargula) hilgendorfii *was reported in 1966 [77] (*Fig. 2.3*). Cypridinid BL reaction requires only three components: luciferin, oxygen, and luciferase [78]. Unlike *Cypridina*, glowing species from other families of Ostracoda, such as Halocypridoidea and Conchoecia, have coelenterazine-dependent luciferases, which is probably related to the fact that their luciferases are not secreted [79].

*C. hilgendorfii *luciferase was cloned in 1989 [80], whereas a successful cloning of *C. noctiluca *luciferase happened much later, in the early 2000s [81]. Luciferases from *Cypridina *(CLuc) are secreted proteins with molecular weight around 62 kDa, which places them among the largest known luciferases. These luciferases exhibit no significant homology with other known luciferases. However, comparison of amino acid sequences of two *Cypridina *luciferases showed a high degree of homology between them (~84%). Nevertheless, the activity of *C. noctiluca *luciferase was much higher than that of *C. hilgendorfii *in experiments with eukaryotic cell cultures [81]. *Cypridina *BL peaks in the range of 448–463 nm, and the reaction demonstrates relatively high quantum yield (0.31) [82]. The BL spectrum depends on ionic strength of solution and is almost pH-independent. The temperature optimum of the reaction is 30°C. A reaction catalyzed by CLuc is strongly inhibited upon addition of EDTA, which probably indicates the involvement of divalent metal ions, such as calcium and magnesium, in the process. Presence of 16 disulfide bonds in CLuc makes their expression in prokaryotic systems almost impossible. However, recently it was shown that production of the enzymes in plant cell cultures is feasible [83], but the presence of two N-glycosylation sites in the protein structure might have an effect on its properties upon expression in eukaryotic cells [83, 84].

Thus, cypridinid luciferases are very stable, allow long-term storage at room temperature, and demonstrate highest quantum yield among all known luciferases. Additionally, they are secreted enzymes, which make them highly suitable for *ex vivo* analysis of intracellular processes. However, extreme instability of *Cypridina* luciferin and its high cost are serious obstacles in the use of cypridinid luciferases in practical applications.

## 4. BACTERIAL LUCIFERASES

The first evidence of light emission by live bacteria was found by Harvey in the early 1920s [85]. Further studies showed that a number of components were necessary for the BL of bacteria, namely FMNH_2_, an aliphatic aldehyde, luciferase, and oxygen. Even though the bacterial luciferin – dodecanal – is oxidized in the course of BL reaction (*Fig. 2.4*), it is not the actual light-emitter. The actual light-emitter in the reaction is luciferase-bound hydroxyflavin. Dodecanal can be replaced by other long-chain aliphatic aldehydes *in vitro* [86]. The maxima of bacterial BL *in vitro *for most of the strains lie within the range of 472–505 nm.

The bioluminescent systems of bacteria *Vibrio harveyi, V. fischeri, Photorhabdus (Xenorhabdus) luminescens, Photobacterium phosphoreum*, and *P. leiognathi* are the most studied to date [85]. All the currently known bacterial luciferases have similar structure, which includes heterodimeric complexes composed of two subunits – α-subunit with the molecular weight of 40 kDa and β-subunit with the molecular weight of 35 kDa. The active site of the enzyme was shown to be located on the α-subunit [87]. Each subunit of luciferase is encoded by a separate gene – *luxA *encodes the α-subunit and *luxB *encodes the β-subunit. These genes were cloned for the first time at the end of the twentieth century [88, 89]. Individual subunits have practically no luciferase activity, and simple mixing them in solution does not restore it [90]. However, the BL activity is restored upon joint renaturation of both recombinant polypeptides [91].

**Fig. 5:**
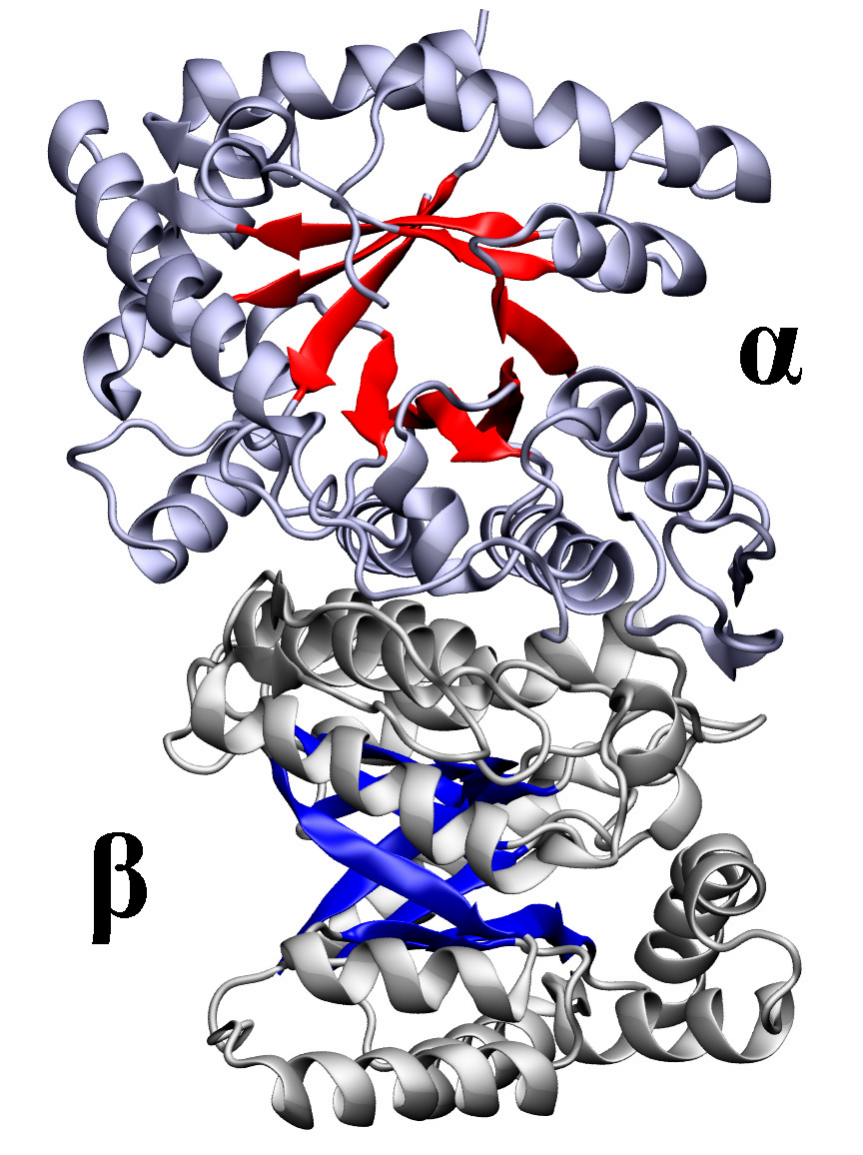
Structure of bacterial luciferase from *V. harveyi*

*V. harveyi *luciferase was crystallized and its X-ray structure was determined (*Fig. 5*) [92, 93]. Both subunits of the luciferase have a similar structure and each of them has one domain containing a β/α-barrel motif. The fragment of α-subunit polypeptide chain forms a mobile loop from phenylalanine 272 to threonine 288, which changes conformation upon binding of FMNH_2_ and protects the latter from non-specific interactions [94]. In addition, conserved histidine 44, aspartic acid 113, and arginine 107 of α-subunit were shown to be crucial for binding of FMNH_2_ and for high quantum yield of the reaction [87, 93, 95, 96]. However, none of the domains, specific for almost all flavin-binding enzymes containing a similar β/α-barrel structure, was found in the structure of bacterial luciferase. This observation probably explains the fact that the protein uses FMNH_2_ as a substrate, and not as a prosthetic group [97].

Luciferases from *P. phosphoreum *and *V. fischeri *are active over a broad pH range from pH 6.0 to 8.0 [98, 99]. Biochemical properties of bacterial luciferases can be significantly improved in mutant forms. For example,* V. fischeri *luciferase is stable at 30°C; however, it loses its activity upon heating to 37°C [100]. *V. harveyi *luciferase, on the contrary, is stable at 37°C. Currently, luciferase from *P. luminescens *is the most frequently used luciferase for imaging bacteria because of its broader temperature stability profile; it remains stable up to 42°C [101].

The encoding of the bacterial BL system in *lux *operon has always been its main advantage over other systems. The operon *luxCDABE *encodes luciferase (*luxA *and *luxB*) and proteins (reductase, transferase, and synthetase) needed for the synthesis of a substrate (*luxCDE*) [102]. For the moment, structures of numerous* lux *operons are known, and each of them can be used for biotechnological applications. The *lux *operon is mainly used in bacterial cells to create biosensors (as a reporter gene) to study the development of bacterial infectious diseases, and for analysis of ecotoxicity. The use of bacterial operon allows to successfully transfer the BL phenotype to different non-luminescent bacterial strains such as *E. coli*, *P. aeruginosa*, *S. typhimurium*,* L. monocytogenes, S. aureus*, *S. pneumoniae* [103-107]. Interestingly, the bioluminescent species of anaerobic bacteria *C. perfringens *and *B. breve *were successfully labeled with *lux*, and BL signal was detected in the intestines of experimental animals in the conditions of extremely low oxygen concentration [108, 109].

Bacterial *lux *genes have been optimized for eukaryotic cells [110] and, in particular, for mammalian cells [111], although the large size of operon, low brightness of luminescence, and cytotoxicity of bacterial luciferin complicate its implementation in heterologous systems. Recently, due to additional changes in the operon and tuning of optimal gene expression, a new “*co Lux*” cassette has been developed, the luminescence of which in HEK293 cells was comparable to that of firefly luciferase [112]. Toxic effect of aliphatic aldehyde in “*co Lux*” was not observed.

## 5. FUNGAL LUCIFERASES

Though first studies of fungal BL began in the 17^th^ century, the structure of fungal luciferin was elucidated only five years ago [113]. Fungal BL reaction is based on luciferin (belonging to styrylpyrone subclass of polyketides), which is formed in two steps from caffeic acid, a common metabolite (*Fig. 2.5*). Fungal luciferases from several species were recently cloned, and one of them from *Neonothopanus nambi *(nnLuz) was successfully applied in various imaging experiments [114]. nnLuz consists of 267 amino acids and has a molecular weight of about 28.5 kDa. The optimum conditions for recombinant nnLuz are pH around 8.0 and temperature below 30°C. When expressed in *P. pastoris *cells, nnLuz was associated with the microsomal fraction and emitted green light with the BL maximum at 520 nm and emission spectrum identical to that of *N. nambi* mycelium. *nnLuz *has been successfully tested as a reporter gene in various heterologous systems, such as *P. pastoris*, early *Xenopus laevis *embryos, human cells, as well as in a whole-body imaging setup of tumor xenografts in mice. The genes of nnLuz and three other enzymes, involved in the luciferin biosynthetic cascade, are members of a gene cluster conserved among the bioluminescent fungi. It has also been shown that introduction of *nnLuz *together with the genes of fungal luciferin biosynthesis into host genomes resulted in yeast cells and even whole plants that were autonomously bioluminescent [115]. The structure of fungal luciferin allows it to be synthesized by a simplified scheme and to be modulated to develop new analogs with improved spectral characteristics [116].

## CONCLUSIONS

**Fig. 6:**
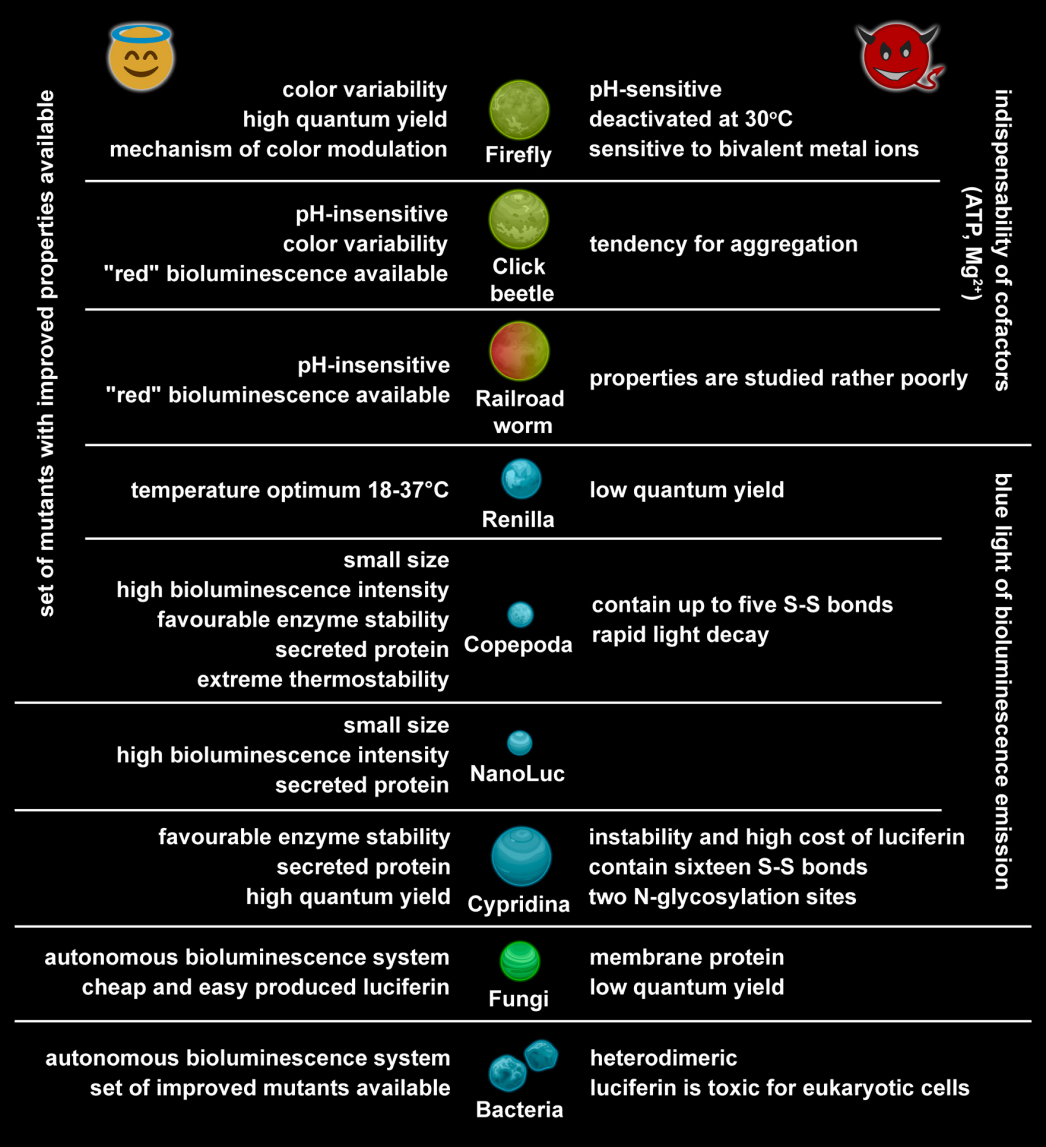
The main, practically significant advantages and disadvantages of natural luciferases

A wide palette of cloned luciferases and their mutant forms provides an excellent opportunity for the practical application of these enzymes in science and medi cine. Despite the large number of existing applications, all the proteins mentioned still have the potential to be used in new approaches or to be used for improving the existing ones. Each luciferase has its own set of drawbacks, but sometimes the limitation for one method is an advantage for another. There is no universal advice on selection of a luciferase for development of new analytical methods, but a few key parameters should be taken into account, such as thermostability, pH optimum of the reaction, and luminescent emission maximum (*Fig. 6*). The authors hope that this review will help researchers in choosing an enzyme to solve a specific problem. There are several dozens of less studied bioluminescent systems that were considered to be out of the scope of the review. Their studies are likely to significantly expand the existing possibilities of applications of bioluminescence in biomedicine.

## Abbreviations

BL: bioluminescence/bioluminescent
BRET: bioluminescence resonance energy transfer
CHO: Chinese hamster ovary cells (cell line)
DLSA: 5’-O-[(N-dehydroluciferyl)-sulphamoyl]-adenosine
GFP: green fluorescent protein
